# Seeking a viable perioperative preemptive analgesia regimen: a prospective, randomized, controlled, double-blind superiority study of intravenous acetaminophen in children undergoing adenotonsillectomy

**DOI:** 10.3389/fped.2026.1844038

**Published:** 2026-06-23

**Authors:** Zugang Yi, Runxin Qiu, Qianyi Qiu, Yiyi Li, Xinyan Gan, Xiang Li, Fang Chen

**Affiliations:** 1 Department of Anesthesiology, Shenzhen Children's Hospital, Shenzhen, China; 2 Health Science Center, Shenzhen University, Shenzhen, China; 3 School of Medicine, Southern University of Science and Technology, Shenzhen, China

**Keywords:** acetaminophen, adenotonsillectomy, emergence delirium, multimodal analgesia, postoperative pain

## Abstract

**Objective:**

To evaluate the analgesic efficacy and safety of pre-induction intravenous acetaminophen in preschool children undergoing day-case adenotonsillectomy.

**Methods:**

A prospective, randomized, double-blind, placebo-controlled trial was conducted in 80 children aged 3–6 years. Participants received 15 mg/kg intravenous acetaminophen or saline 15 min prior to anesthetic induction. The primary outcome was FLACC pain score at 1 h post-extubation. Secondary outcomes included FLACC scores at other postoperative time points, PAED emergence delirium scores, PACU opioid rescue rates, home analgesia use, and adverse events within 24 h.

**Results:**

At 1 h post-extubation, FLACC scores were significantly lower in the acetaminophen group [median difference −1.0, 95% CI −2.0 to −1.0; *P* = 0.002]. PACU opioid rescue rates were reduced (20.0% vs. 45.0%; absolute risk difference −25%, 95% CI −44.6 to −5.4%; RR = 0.44; NNT = 3.3). No significant differences were observed in PAED scores, home analgesia use, or 24-hour adverse events. Early postoperative analgesic benefit was time-limited, with no significant differences at 4, 8, or 24 h postoperatively.

**Conclusions:**

Pre-induction intravenous acetaminophen effectively reduces early postoperative pain and PACU opioid requirements without increasing adverse events in preschool children undergoing day-case adenotonsillectomy. Emergence delirium was unaffected in this low-risk population. The findings support its use as a foundational component of multimodal analgesia strategies in pediatric day-case adenotonsillectomy.

**Clinical Trial Registration:**

https://www.chictr.org.cn/showproj.html?proj=213700, ChiCTR2400079538.

## 1 Introduction

Adenotonsillectomy is among the most commonly performed elective procedures in pediatric otolaryngology. It is primarily indicated for the treatment of obstructive sleep apnea and recurrent suppurative tonsillitis in children (1, 2). The removal of the tonsils induces an inflammatory cascade that facilitates healing, while also leaving open wounds in the oropharynx. The glossopharyngeal and trigeminal nerves are thereby exposed (3). Consequently, the procedure is associated with significant postoperative pain. Pain peaks are typically observed during anesthetic emergence, which may provoke crying, agitation, and feeding refusal, thereby increasing the risk of perioperative complications and delaying discharge. Pediatric perioperative analgesia is often delivered using multimodal strategies, including opioids, nonsteroidal anti-inflammatory drugs (NSAIDs), and local anesthetics. Although opioids provide effective analgesia, they can induce respiratory depression, nausea, vomiting, and excessive sedation in children (4). NSAIDs may reduce opioid requirements but can increase the risk of surgical site bleeding. In contrast, acetaminophen exerts analgesic and antipyretic effects through central cyclooxygenase inhibition. Platelet function is not affected, and its safety profile is high. It is recommended by the PROSPECT guidelines as a foundational analgesic for pediatric tonsil surgery (5).

Previous studies have demonstrated that acetaminophen can reduce postoperative pain scores and opioid consumption. However, these studies were often limited by small sample sizes, wide age ranges, or non-day surgery settings, and intravenous administration was rarely employed (5–9). In contrast, the present study administered acetaminophen intravenously prior to anesthetic induction in preschool children undergoing day-case adenotonsillectomy. Intravenous delivery allows rapid achievement of peak plasma concentrations, precisely covering the early postoperative pain peak, thereby enabling a reliable assessment of preemptive analgesic effects and opioid-sparing outcomes in the post-anesthesia care unit (PACU).

Based on this rationale, a prospective, randomized, double-blind, placebo-controlled design was adopted. The study aimed to evaluate whether pre-induction intravenous administration of acetaminophen could reduce early postoperative pain, decrease opioid rescue requirements in the PACU, and maintain perioperative safety, thereby providing evidence-based guidance for perioperative analgesia in preschool children undergoing day-case adenotonsillectomy.

## 2 Methods

### 2.1 Study design and ethical approval

This study was conducted as a single-center, prospective, randomized, double-blind, placebo-controlled clinical trial at the Day Surgery Center of Shenzhen Children's Hospital. The study protocol was approved by the Ethics Committee of Shenzhen Children's Hospital (approval number: 202314202). Written informed consent was obtained from the legal guardians of all participants prior to surgery. The trial was registered with the Chinese Clinical Trial Registry（ChiCTR2400079538）. The study was conducted in strict accordance with the Declaration of Helsinki and the CONSORT 2010 guidelines for reporting randomized controlled trials.

### 2.2 Study population

Inclusion criteria were as follows: (i) age 3–6 years (preschool children) scheduled for elective day-case adenotonsillectomy; (ii) American Society of Anesthesiologists (ASA) physical status I–II; and (iii) written informed consent provided by the child's legal guardian.

Exclusion criteria included: (i) allergy to acetaminophen, opioids, or any anesthetic agents used in this study; (ii) history of chronic pain or long-term use of analgesic medications; (iii) hepatic or renal dysfunction within one month prior to surgery; (iv) coagulopathy or use of anticoagulant/antiplatelet agents before surgery; (v) active infection such as upper respiratory tract infection or fever prior to surgery; and (vi) neurodevelopmental disorders preventing cooperation with pain and emergence delirium assessments.

### 2.3 Randomization and blinding

Randomization was performed using a computer-generated random number table, assigning participants in a 1:1 ratio to either the acetaminophen group (*n* = 40) or the placebo group (*n* = 40). The randomization sequence was generated by an independent staff member not involved in anesthesia administration, outcome assessment, or data analysis. Allocation concealment was maintained using sequentially numbered, opaque, sealed envelopes, which were opened and the study drugs prepared only by a dedicated nurse not involved in perioperative care. Participants in the acetaminophen group received an intravenous infusion of 15 mg/kg acetaminophen 15 min before anesthetic induction. Participants in the placebo group received an equal volume of 0.9% saline at the same time point. The appearance, formulation, and method of infusion were identical for both solutions to ensure blinding. The study was conducted under double-blind conditions, with participants, guardians, anesthesiologists, surgeons, PACU nurses, outcome assessors, and data analysts blinded to group allocation until study completion and database lock.

### 2.4 Perioperative management

All participants were fasted for 8 h and abstained from fluids for 2 h prior to surgery. Peripheral intravenous access was established, and intramuscular atropine (0.01 mg/kg) was administered preoperatively. Upon arrival in the operating room, standard monitoring was applied, including electrocardiography (ECG), noninvasive blood pressure (NIBP), heart rate (HR), oxygen saturation (SpO₂), and end-tidal carbon dioxide (PETCO₂).

Following the infusion of acetaminophen or saline, anesthesia induction was initiated with propofol (2.5 mg/kg), fentanyl (2 μg/kg), and rocuronium (0.6 mg/kg). Tracheal intubation was performed after loss of consciousness and adequate neuromuscular relaxation. Mechanical ventilation was set with a tidal volume of 6–8 mL/kg, and respiratory rate was adjusted to maintain PETCO₂ between 35 and 45 mmHg. Anesthesia was maintained with 1.5%–2.0% sevoflurane in a 50% oxygen–air mixture, with depth titrated according to intraoperative bispectral index (BIS) monitoring.

All participants received intraoperative intravenous dexamethasone and tropisetron (0.1 mg/kg). Surgery was performed by the same team of experienced otolaryngologists, using standard electrocautery dissection for tonsillectomy and radiofrequency ablation for adenoidectomy. Hemostasis methods were standardized across all cases. At the end of surgery, all anesthetic agents were discontinued, and participants were transferred to the post-anesthesia care unit (PACU) with the endotracheal tube in place. Neostigmine (0.02 mg/kg) and atropine (0.01 mg/kg) were administered intravenously to reverse residual neuromuscular blockade. Tracheal extubation was performed once the patient met standard criteria, followed by at least 10 min of observation in the PACU. Participants were then transferred to the day surgery ward, with discharge readiness evaluated at 4 h postoperatively. Post-discharge follow-up was conducted via telephone.

Post-discharge analgesia comprised ibuprofen suspension administered by caregivers on an as-required basis. Caregivers were instructed to give ibuprofen only if the child self-reported pain or if the caregiver independently judged that the child exhibited pain-related behaviours (e.g., crying, irritability, refusal to feed). Dosing was weight-based: 4 mL (10–15 kg), 5 mL (16–21 kg), or 8 mL (22–27 kg) per administration, with repetition permitted. Time to first administration and the proportion of children requiring home analgesia were recorded as secondary outcomes.

### 2.5 Outcome measures

The primary outcome measure was the FLACC pain score at 1 h after extubation (T1). The FLACC scale is an internationally recognized tool for assessing pain in children aged 2 months to 7 years. It evaluates five domains: facial expression, leg movement, activity, cry, and consolability. Each domain is scored from 0 to 2, yielding a total score of 0–10, with higher scores indicating greater pain intensity. A score of 0 represents no pain, 1–3 indicates mild pain, 4–6 moderate pain, and 7–10 severe pain.

Secondary outcome measures included: (i) FLACC pain scores at 10 min (T0), 4 h (T2), 8 h (T3), and 24 h (T4) post-extubation; (ii) Pediatric Anesthesia Emergence Delirium (PAED) scores at T0, T1, and T2. The PAED scale assesses eye contact, purposeful actions, awareness of surroundings, restlessness, and consolability, with total scores ranging from 0 to 20, where higher scores indicate more severe delirium; (iii) the rate of fentanyl rescue analgesia in the PACU, defined as the proportion of children with a FLACC score >4 sustained for ≥1 min who received intravenous fentanyl 0.25 μg/kg as judged by the attending PACU physician; (iv) the time to first home analgesic administration, defined as the interval from extubation to the first oral dose of ibuprofen post-discharge; and (v) the incidence of adverse events within 24 h postoperatively, including nausea, vomiting, abdominal pain, pruritus, respiratory depression, surgical site bleeding, and unplanned readmission.

FLACC scores at 8 and 24 h were obtained exclusively by telephone follow-up, not by in-person assessment. Prior to discharge, caregivers received brief training and a standardized instruction leaflet on FLACC scale use. During the telephone call, a single trained research nurse administered a structured questionnaire covering the five standard domains (facial expression, legs, activity, cry, consolability). Caregivers selected from graded descriptors for each item, and the nurse guided scoring to ensure consistent application, generating domain scores of 0–2.

### 2.6 Data collection

Data collection and scale assessments were performed by nurses who received standardized training and remained blinded to group allocation throughout the study. Preoperative baseline demographic data were collected, including age, sex, weight, and ASA physical status. Intraoperative data recorded included surgical duration, anesthesia duration, and PACU stay time, along with documentation of fentanyl rescue analgesia in the PACU. Postoperative follow-up was conducted at predetermined time points to assess FLACC pain scores, PAED emergence delirium scores, home analgesic usage, and the incidence of adverse events within 24 h.

### 2.7 Sample size and statistical analysis

The sample size was calculated based on the primary outcome, the FLACC pain score at T1. Preliminary data indicated that children who did not receive preoperative acetaminophen had a mean FLACC score of 4.0 ± 1.5 at 1 h postoperatively. Pre-induction acetaminophen was expected to reduce the score by 1.5 points. Using a two-sided independent-samples t-test with a significance level of *α* = 0.05 and a power of 90% (1−β), 34 participants per group were required. Accounting for a 10% dropout rate, 40 participants were planned for inclusion in each group, yielding a total of 80 participants.

Statistical analyses were conducted using SPSS version 15.0. A two-sided *P* value <0.05 was considered statistically significant. Continuous variables were first assessed for normality using the Shapiro–Wilk test. Normally distributed data were presented as mean ± standard deviation (X ± s) and compared between groups using an independent-samples t-test. Non-normally distributed data were reported as median (interquartile range, IQR) and compared using the Mann–Whitney U test. Categorical variables were expressed as frequency (percentage, *n*%) and analyzed using the chi-square test or Fisher's exact test, as appropriate.

## 3 Results

A total of 82 children meeting the inclusion criteria were screened, of whom 2 were excluded due to missing postoperative follow-up data. Ultimately, 80 participants completed the study and were included in the final analysis, with 40 in the acetaminophen group and 40 in the placebo group. Baseline demographic and perioperative characteristics were comparable between groups, with all participants classified as ASA II. The overall mean age was 58.01 ± 8.92 months, 66.3% were male, and the mean weight was 17.95 ± 3.03 kg. Mean surgical duration was 15.95 ± 4.83 min, and mean PACU stay was 22.61 ± 6.54 min. No statistically significant differences were observed between groups in age, sex, weight, ASA classification, surgical duration, or PACU stay (all *P* > 0.05), as detailed in Table 1.

**Table 1:** Baseline characteristics of children in the two groups [x ± s/n(%)].

Characteristics	Overall (*n* = 80)	Acetaminophen group (*n* = 40)	Placebo group (*n* = 40)	*P* ^a^ ^,^ ^b^
Age (months)	58.01 ± 8.92	58.18 ± 8.76	57.85 ± 9.19	0.872^a^
Male	53 (66.3%)	27 (67.5%)	26 (65%)	0.813^b^
Body weight (kg)	17.95 ± 3.03	18.09 ± 3.16	17.80 ± 2.94	0.677^a^
Operation duration (min)	15.95 ± 4.83	15.75 ± 4.95	16.15 ± 4.76	0.714^a^
PACU stay duration (min)	22.61 ± 6.54	23.43 ± 7.46	21.80 ± 5.46	0.269^a^

X ± s, mean ± standard deviation; *n* (%), frequency (percentage).

a comparison between groups was performed using an independent-samples t-test.

b comparison was performed using the chi-square test or Fisher's exact test, as appropriate.

The primary outcome was the FLACC pain score at 1 h post-extubation (T1). Median FLACC scores at T1 were lower in the acetaminophen group compared with the placebo group, with a median difference of −1.0 (95% CI −2.0 to −1.0), which was statistically significant (Z = 3.166, *P* = 0.002). At other time points, median FLACC scores at 10 min post-extubation (T0) were also lower in the acetaminophen group than in the placebo group, with a median difference of −1.0 (95% CI −2.0 to −1.0), reaching statistical significance (Z = 2.955, *P* = 0.003). No significant differences were observed at 4 h (T2), 8 h (T3), or 24 h (T4) post-extubation (all *P* > 0.05), as shown in Table 2.

**Table 2:** Various observation indicators of children in the two groups at different time points [M(IQR)/*n*(%)].

Time point	Acetaminophen group (*n* = 40)	Placebo group (*n* = 40)	Absolute risk difference (%)/Median difference (95%CI)	P^b^^,^^c^
Primary outcome measure FLACC score				
T0	2.0 (1–4)	4 (2–6)	−1 (−2, −1)	0.003^c^
T1	2 (0–3.75)	3 (2–5)	−1 (−2, −1)	0.002^c^
T2	0 (0–2)	1 (0–2)	0.0 (−1, 0)	0.095^c^
T3	1 (0–2)	1 (0–2)	0 (0, 1)	0.62^c^
T4	1 (0–1)	1 (0–1)	0 (0, 0)	0.93^c^
Secondary outcome measure PAED score				
T0	5 (4–6)	5 (4–8)	−1 (−2, 0)	0.249^c^
T1	2 (0–3)	2 (2–4)	0 (−1, 0)	0.237^c^
T2	0 (0–1)	1 (0–1)	0 (0, 0)	0.187^c^
PACU rescue analgesia rate	8 (20%)	18 (45%)	−25 (−44.6, −5.4)	0.017^b^
Home analgesia rate	13 (32.5%)	16 (40.0%)	−7.5 (−28.5, 13.5)	0.485^b^
Time to first home analgesia	476 (454–516)	521 (417–562)	−14 (−74, 49)	1.000^b^
Incidence of adverse events within 24 h after surgery				
Postoperative nausea and vomiting	1 (2.5%)	0 (0%)	2.5 (−2.3, 7.3)	1.000^b^
Postoperative abdominal pain	1 (2.5%)	1 (2.5%)	0 (−6.8, 6.8)	1.000^b^
Other adverse reactions (respiratory depression, pruritus, readmission, etc.)	0 (0%)	0 (0%)	0	1.000^b^

IQR, interquartile range; CI, confidence interval; FLACC (Face, Legs, Activity, Cry, Consolability Scale), a standardized tool for pediatric pain assessment; PAED (Pediatric Anesthesia Emergence Delirium scale), a scale for evaluating emergence delirium in children; PACU, post-anesthesia care unit.

b comparisons were performed using the chi-square test or Fisher's exact test.

c non-normally distributed data were compared using the Mann–Whitney U test.

At 10 min (T0), 1 h (T1), and 4 h (T2) post-extubation, median PAED scores did not differ significantly between the acetaminophen and placebo groups (all *P* > 0.05).

During PACU stay, 8 children (20.0%) in the acetaminophen group required fentanyl rescue analgesia, compared with 18 children (45.0%) in the placebo group. The acetaminophen group had a significantly lower rate of PACU fentanyl rescue, with an absolute risk difference of −25% (95% CI −44.6% to −5.4%), which was statistically significant (*χ*² = 5.698, *P* = 0.017). The relative risk associated with the intervention was 0.44 (95% CI 0.217–0.894), and the number needed to treat (NNT) was 3.3.

Post-discharge home analgesia was required by 13 children (32.5%) in the acetaminophen group and 16 children (40.0%) in the placebo group. No statistically significant difference was observed between groups in home analgesia rates (*χ*² = 0.487, *P* = 0.485), with an absolute risk difference of −7.5% (95% CI −28.5% to 13.5%). Among children who required home analgesia, the median time to first home dose did not differ significantly between groups (Z100/P100 = 1.000), with a median difference of −14.0 min (95% CI −74.0–49.0).

Within 24 h postoperatively, no significant differences were observed in adverse event rates between groups (all *P* > 0.05). In the acetaminophen group, one child (2.5%) experienced nausea and vomiting, and one child (2.5%) experienced abdominal pain. In the placebo group, one child (2.5%) experienced abdominal pain, and no cases of nausea or vomiting occurred. Neither group experienced serious adverse events requiring special intervention, including respiratory depression, active surgical site bleeding, pruritus, or unplanned readmission, as detailed in Table 2.

## 4 Discussion

This study employed a prospective, randomized, double-blind, placebo-controlled design to evaluate the analgesic efficacy and safety of pre-induction intravenous administration of 15 mg/kg acetaminophen in preschool children aged 3–6 years undergoing day-case adenotonsillectomy. The results demonstrated a median FLACC score difference of 1 point at 1 h post-extubation (*P* = 0.002), indicating a significant benefit in early postoperative pain control for the intervention group. Concurrently, the rate of opioid rescue analgesia in the PACU was significantly reduced, reflecting the clinical analgesic effect of acetaminophen. The intervention did not increase the risk of perioperative adverse events; however, it did not significantly reduce emergence delirium scores in children. This study provides high-quality, single-center randomized controlled trial (RCT) evidence supporting a multimodal perioperative analgesia framework for day-case adenotonsillectomy in preschool children and contributes data for the multimodal application of pediatric day-surgery analgesia guidelines.

Pharmacologically, acetaminophen reduces the synthesis and release of prostaglandin E2 by inhibiting central COX-2 pathways and downregulates the activity of descending pain facilitation pathways, thereby blocking central sensitization induced by intraoperative nociceptive stimuli. Peak plasma concentrations are achieved within minutes after intravenous administration. In this study, acetaminophen was administered 15 min prior to anesthetic induction, allowing effective drug concentrations to be present at the time of surgical incision and tissue injury, thereby precisely covering the postoperative pain peak within 0–2 h and achieving early analgesic effects.

At 1 h post-extubation, FLACC scores differed significantly between groups [2 (0–3.75) vs. 3 (2–5), 95% CI −2.0 to −1.0, *P* = 0.002], consistent with the findings of similar RCTs reported by Rachel and Toshiaki (10, 11). However, the type and half-life of intraoperative opioids may influence this outcome. In this study, short-acting opioids (e.g., fentanyl) were used intraoperatively and for postoperative rescue, providing limited early postoperative analgesia. As the effect of intraoperative opioids waned, the pre-induction intravenous acetaminophen gradually exerted its analgesic effect, resulting in a significant reduction in PACU rescue opioid requirements. These observations highlight that the pharmacologic properties of intraoperative opioids are an important factor in interpreting early postoperative pain scores. The use of long-acting, potent opioids (e.g., sufentanil, morphine) could potentially mask the additional effect of acetaminophen at 1 h post-extubation, thereby diminishing observable differences. Consequently, the results demonstrate both the early analgesic efficacy of acetaminophen and the critical influence of intraoperative opioid type and half-life on early pain assessment.

The analgesic effect of the intervention was time-dependent. Significant benefit was observed within 0–1 h postoperatively, whereas no statistically significant differences were detected at 4 h (T2), 8 h (T3), or 24 h (T4) post-extubation. A single 15 mg/kg intravenous dose of acetaminophen has a relatively short half-life, typically providing analgesia for 4–6 h; by 8 h postoperatively, plasma concentrations fall below the effective threshold, and analgesic efficacy diminishes over time. The natural course of surgical trauma also influences pain scores, as the acute pain peak after adenotonsillectomy is concentrated from emergence to 2 h postoperatively, with pain and inflammatory responses declining naturally after 4 h, reducing intergroup differences over time. Methodological factors may additionally affect longer-term pain assessments: although standardized evaluations were performed by trained nurses, FLACC scores at 8 and 24 h were collected via telephone follow-up with caregivers, potentially influenced by subjective judgment, home environment, feeding, activity, and consolability, which may obscure subtle differences. Importantly, the core population of this study consisted of day-surgery patients, where the clinical focus is on smooth emergence and minimizing delayed discharge. Postoperative pain beyond the early period was generally mild, and the intervention effectively targeted the high-risk window for early postoperative pain, achieving the predefined clinical objective.

The sample size calculation in this study was based on a prespecified minimal clinically important difference (MCID) of 1.5 points. The observed median difference was 1 point; however, the statistical analysis yielded *P* = 0.002. Although the difference was smaller than the prespecified MCID, it remained statistically significant, potentially due to lower-than-anticipated data variability or a sample size sufficient to detect smaller differences. Clinically, the lower bound of the 95% confidence interval was −2.0, exceeding the MCID threshold, indicating that the true effect size meets the standard for clinical importance. Notably, this 1-point difference corresponded to PACU opioid usage rates of 45% in the intervention group and 20% in the control group, demonstrating a substantial opioid-sparing effect of significant clinical value.

The rate of fentanyl rescue analgesia in the PACU was reduced by an absolute 25% in the intervention group compared with the control group (95% CI −44.6% to −5.4%), corresponding to a 56% relative risk reduction (RR = 0.44, 95% CI 0.217–0.894) and a number needed to treat (NNT) of 3.3; that is, one opioid exposure was prevented for every three children receiving the intervention. This finding holds substantial clinical significance in the management of pediatric day-case surgery.

First, perioperative safety was markedly improved. Opioids are major risk factors for respiratory depression, postoperative nausea and vomiting, emergence agitation, and excessive sedation in children (12–14). In day-case patients, residual opioid effects can increase post-discharge care risks and contribute to unplanned readmissions (15). Consistent with the findings of Gerdien et al., intermittent intravenous acetaminophen in infants aged 0–3 years undergoing cardiac surgery reduced median weight-adjusted morphine consumption over 48 h by 79% (16). In the present study, nearly half of the children avoided additional opioid exposure, thereby reducing the risk of opioid-related perioperative adverse events and aligning with ERAS core principles.

Second, multimodal analgesic strategies were optimized. A single pre-induction dose of acetaminophen achieved substantial opioid-sparing effects without the need for NSAIDs, which carry bleeding risks, providing a safe, simple, and cost-effective analgesic regimen for pediatric tonsil surgery that can be implemented in resource-limited settings. No significant differences in adverse events within 24 h postoperatively were observed between groups; neither respiratory depression nor surgical site bleeding occurred. These findings further confirm the safety of 15 mg/kg intravenous acetaminophen in preschool children, in accordance with recommendations from international pediatric anesthesiology guidelines.

Post-discharge home analgesia rates and time to first home analgesic administration did not differ significantly between groups, consistent with the negative findings for pain scores at 8 and 24 h postoperatively. This is attributable to the clinical analgesic effect of a single 15 mg/kg intravenous dose of acetaminophen, which typically lasts 4–6 h; by the time of discharge, drug metabolism is nearly complete, and thus home analgesic requirements are unaffected. Furthermore, residual effects from intra-hospital opioid rescue in the placebo group also contributed to similar post-discharge pain baselines between groups. These observations indicate that a single pre-induction dose of acetaminophen does not interfere with standardized home analgesia management.

The analysis of postoperative emergence delirium revealed no statistically significant differences in PAED scores between groups at T0, T1, and T2. The study's sample size was calculated based on the primary endpoint of pain scores, limiting the power to detect differences in secondary outcomes such as emergence delirium, and the study population was overall low-risk (median PAED scores up to 5, FLACC scores mild to moderate). Notably, a previous Turkish randomized controlled trial (Keles et al.) demonstrated that preemptive analgesia significantly reduced PAED scores at the specific 5-minute postoperative time point, indicating that population characteristics, timing of administration, and study design may influence the detection of emergence delirium. These findings underscore the importance of study design and patient selection when interpreting negative results in low-risk pediatric populations (17). Previous research indicates that only moderate to severe pain substantially increases the risk of postoperative emergence delirium in children (18–21), whereas mild to moderate pain is insufficient to drive significant changes. Emergence delirium is a multifactorial neurobehavioral outcome, influenced by preoperative anxiety, child temperament, neurodevelopmental status, and parental consolability (22–24). Even with balanced baseline characteristics between groups, potential effects of acetaminophen may be obscured.

This study further expands the evidence base for intravenous acetaminophen as a perioperative analgesic in children. The core findings are consistent with prior high-quality RCTs: Keles et al. reported that in children aged 3–7 years undergoing full-mouth dental rehabilitation under general anesthesia, preoperative intravenous administration of 15 mg/kg acetaminophen resulted in significantly lower Wong-Baker Faces Pain Scale scores in the post-anesthesia care unit and at 2, 4, and 8 h postoperatively, along with reduced rescue analgesia requirements and total intravenous fentanyl consumption (25). Similarly, Kim and Rizkalla observed decreased postoperative opioid consumption in adolescent spinal fusion surgery (26, 27).

A systematic review including five RCTs (total 443 children, mean age 2.12 ± 2.81 years) showed no significant differences in pain scores between “intravenous acetaminophen plus opioids” and “opioids alone,” although opioid consumption tended to decrease and the risk of minor adverse events was reduced (28). These studies support both the efficacy and reproducibility of intravenous acetaminophen interventions. Differences in some findings may be attributable to study design: Taylor et al. conducted a retrospective analysis in a pediatric ICU and found that immediate postoperative intravenous acetaminophen did not significantly reduce cumulative opioid use (29); Yousuf et al. reported that administration 1 h before the end of hypospadias repair did not improve pain or sedation scores at 6 h postoperatively (30), indicating that the observation window exceeded the drug's effective duration. Likewise, Kim et al. demonstrated that pre-incision administration was more effective than post-incision administration in reducing postoperative opioid requirements (26).

Evidence regarding postoperative emergence delirium is limited. The DEXACET trial showed that in elderly cardiac surgery patients, co-administration of acetaminophen with propofol or dexmedetomidine reduced delirium incidence (31). In the present study, single-drug acetaminophen intervention did not produce a significant benefit in delirium, suggesting that in low-risk children, analgesia with acetaminophen alone is insufficient to influence emergence delirium. Further high-quality studies are needed to confirm these findings.

The limitations of this study include its single-center design and the restriction to healthy preschool children, which may limit the generalizability of the findings; a sample size insufficient to detect subtle differences in secondary outcomes; the absence of serum drug concentration measurements, precluding analysis of dose–response relationships; and follow-up limited to 24 h, without assessment of long-term postoperative recovery or psychological status. Nonetheless, the study demonstrates that pre-induction intravenous administration of 15 mg/kg acetaminophen effectively reduces early postoperative pain and PACU opioid use without increasing adverse events, providing evidence to support multimodal analgesia strategies for day-case adenotonsillectomy in preschool children and informing future optimization of dosing and timing.

In conclusion, pre-induction intravenous administration of 15 mg/kg acetaminophen significantly reduces early postoperative pain scores and substantially decreases PACU opioid rescue requirements in preschool children undergoing day-case adenotonsillectomy, without increasing the risk of perioperative adverse events. Furthermore, the intervention did not significantly affect emergence delirium scores in this low-risk population. Overall, pre-induction intravenous acetaminophen can serve as a foundational component of a multimodal analgesic strategy for day-case adenotonsillectomy in preschool children, providing evidence-based guidance for clinical practice and informing future optimization of dosing and timing in higher-risk populations.

## Data Availability

The original contributions presented in the study are included in the article/Supplementary Material, further inquiries can be directed to the corresponding author.
